# Silica Nanowires Templated by Amyloid-like Fibrils

**DOI:** 10.1002/anie.201508415

**Published:** 2015-10-05

**Authors:** Zahraa S Al-Garawi, Julian R Thorpe, Louise C Serpell

**Affiliations:** School of Life Sciences, University of Sussex Falmer, BN1 9QG (UK) E-mail: L.C.Serpell@sussex.ac.uk; Chemistry Department, College of Sciences, Al-Mustansyriah University (Iraq)

**Keywords:** amyloid fibrils, cross-β-structure, nanotubes, silica, tetraethylorthosilicate

## Abstract

Many peptides self-assemble to form amyloid fibrils. We previously explored the sequence propensity to form amyloid using variants of a designed peptide with sequence KFFEAAAKKFFE. These variant peptides form highly stable amyloid fibrils with varied lateral assembly and are ideal to template further assembly of non-proteinaceous material. Herein, we show that the fibrils formed by peptide variants can be coated with a layer of silica to produce silica nanowires using tetraethyl-orthosilicate. The resulting nanowires were characterized using electron microscopy (TEM), X-ray fiber diffraction, FTIR and cross-section EM to reveal a nanostructure with peptidic core. Lysine residues play a role in templating the formation of silica on the fibril surface and, using this library of peptides, we have explored the contributions of lysine as well as arginine to silica templating, and find that sequence plays an important role in determining the physical nature and structure of the resulting nanowires.

Molecular self-assembly occurs spontaneously when mole-cules associate with each other to form three-dimensional structures consisting of many weak and reversible interactions such as van der Waals forces, hydrogen bonds, and electrostatic and hydrophobic interactions. All these interactions are essential to maintain thermodynamically stable and well-ordered supramolecular structures.[1] There has been significant interest in the self-assembly of proteins and peptides into β-sheet fibril structures known as amyloid fibrils owing to their association with a growing list of protein misfolding diseases.[2] On the other hand, there has been significant effort to create functional bionanomaterials utilizing self-assembled peptides, such as hydrogels for tissue engineering,[3] scaffolds for vaccine development,[4] drug delivery,[5] and for other effective biological applications.[6] Diverse fibrillar morphologies can be formed, including tapes,[7] tubes,[8] and networks.[9] These versatile structures could be used in nanotechnology devices, such as electronics, catalysis, and sensors, as well as biomedical applications.[10]

Amyloid fibrils are characterized by their cross-β structural core, composed of β-strands associated through hydrogen bonding (4.76 Å) to form β-ribbons running parallel to the fiber axis, and by hydrophobic, aromatic, and electrostatic interactions between nearby side chain groups, with 9.5–10 Å distance between β-sheets.[11]

The key advantages of using amyloidogenic peptides for bionanotechnology arise from their ability to self-assemble, their diverse chemical and physical properties, and formation of highly stable assemblies making them an attractive class of structures for mineralization.[12] Tetraethyl-orthosilicate (TEOS) is a silica precursor that can be used to coat polymers.[13] Several peptides have been investigated to enhance silica formation, and poly-Lys was found to be able to stimulate the hydrolysis of TEOS, forming silicates through its positively charged amino group.[14]

We previously characterized the structure of a self-assembled peptide with the sequence KFFEAAAKKFFE that assembled into laterally associated fibrils. The structure was deciphered using a combination of X-ray fiber diffraction (FD) and electron diffraction.[15] To investigate the sequence contribution to self-assembly, architecture, and morphology, we further investigated the effect of altering the precursor sequence and found that the aromatic F residues were essential for amyloid formation, whilst K contributed to the lateral association of protofilaments and to the inter-sheet spacing and interdigitation of the side chains between β-sheets.[16] Substitution of K for either R or A was used to study the effect of electrostatic interactions on the self-assembled fibrillar structures of amyloid. This work produced a library of closely related self-assembling peptides for further functionalization.

Herein, we characterize the siliconization of a range of sequences based on the original sequence, KFFEAAAKKFFE. Using substitution of K for either A or R, we have gained insights into the silica coating from TEOS, and are able to produce morphologically diverse silica nanowires. Fibers before and after silica coating were characterized using TEM, X ray-FD, and Fourier transform infrared spectroscopy (FTIR), revealing that the nanowires (NWs) retain a cross-β peptide core despite the harsh conditions.

Peptides with the sequences listed in the Supporting Information (Table S1) were self-assembled in water or phosphate buffered saline (PBS) as described in the methods (see the Supporting Information), and their fibrillar nature confirmed using TEM (Figure 1). These peptides have been previously assembled and characterized in PBS and water, and their structures described.[16] Electron micrographs showed fibrils with diameters varying from 8.8 nm–20.6 for K/A. K/R fibrils tend to laterally associate and ranged from 17.5 to 26.6 nm (Supporting Information, Tables S2, S3).

**Figure 1:**
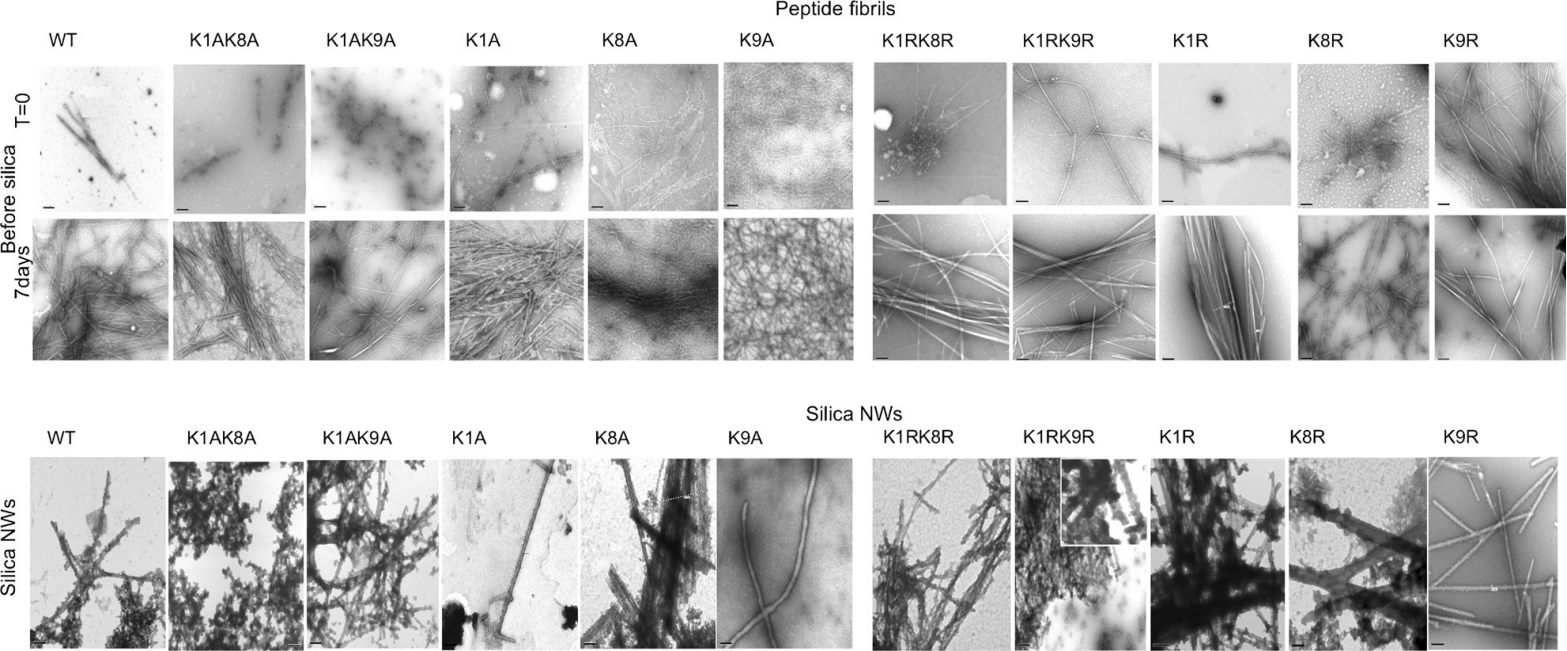
Negative-stain transmission electron micrographs showing the appearance of self-assembled peptides immediately after solubilization, after seven days incubation in water or PBS, and following silica coating. Scale bar=200 nm.

All variants contained E, which is negatively charged at pH 6.0–8.0 owing to its carboxylic group (p*K*_a_ 2.10, and as a side chain 4.07), and two different basic amino acids with different order in basicity (R>K, p*K*_a_ side chains being 10.54 and 12.47, respectively). Orthosilicic acid (silanol group), however, has p*K*_a_ 9.8, which is reduced during polymerization to become negatively charged to produce silica. Because of this, silica coating is markedly dependent on the p*K*_a_ of the amino acids,[17] and we therefore explored the silica coating of the fibrils formed by the library of peptides (Supporting Information, Table S1).

K/A peptide–silica NWs formed a solid and hard white precipitate after 36 h at RT whilst a gel-silica-precipitate was observed for K/R peptide–silica NWs.

Negative-stain TEM was used to examine fibrils following silica coating (Figure 1). All fibrils appeared to have thickened except for those fibrils formed by K1A, K1AK8A, and K9A. For K1A and K9A, very few fibers (less than two per grid) were observed, and for K1AK8A, fibrils could not be observed following TEOS treatment although amorphous silica shells were observed. In contrast, K1AK9A was able to form silica NWs. After TEOS treatment the diameters of the remaining peptide fibrils increased from 8.8–26.6 nm to 48.5–238.6 nm. Silica structures varied in length, and some variants formed bundles of NWs (Figure 1). All silica NWs have increased widths (Supporting Information, Tables S2, S3) and also shared an apparent low electron density central channel (Figure 1).

The amyloid nature of fibrils can be confirmed using X-ray-FD to obtain the characteristic cross-β pattern.[18] All fibrils formed by the WT, K/A, and K/R variants exhibited characteristic cross-β patterns before silica coating (Figure 2). To investigate whether the peptide cross-β core remains in the central channel, X-ray-FD was used to examine the structure of silica NWs. Several of the silica NWs still gave 4.7 Å and 10 Å reflections, supporting the view that the peptide cross-β structure remains within the tubes. However, the equatorial signal, usually found around 10 Å, was not observed for K8A, possibly owing to reduced lateral association of the fibrils in the silica NW samples (Figure 3). These results revealed that the silica NWs retained their original β-sheet conformation.

**Figure 2:**
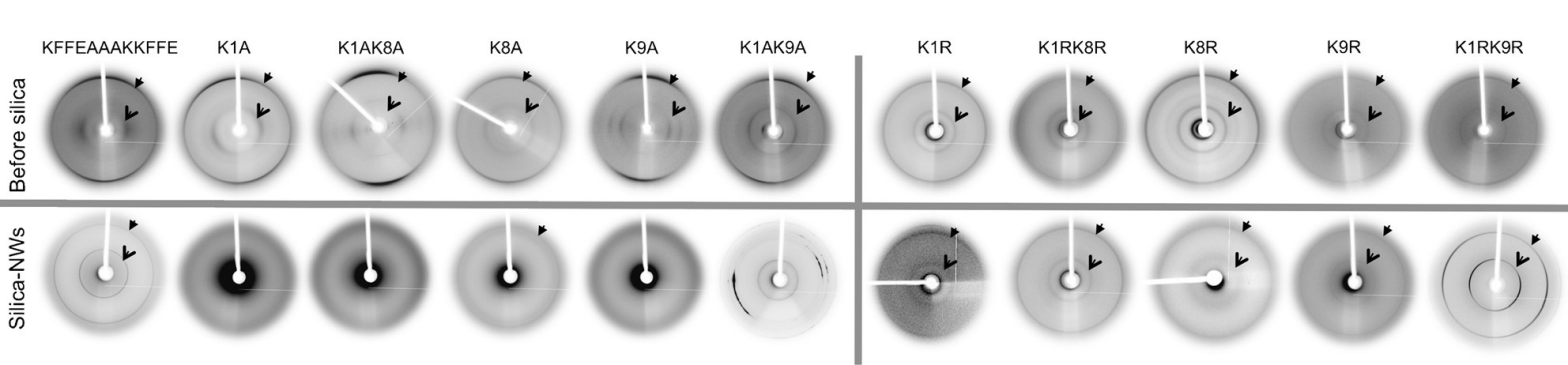
X-ray fiber diffraction patterns for peptide fibrils before and after silica coating. Open black arrows: −10–11 Å equatorial diffraction signal, closed black arrow: 4.7 Å meridional signal, each arising from the cross-β structure of the peptide. All fibrils before silica show the cross-β signals whilst K1A, K1AK8A, K1AK9A, and K9A show no signals following silica coating. K8A silica NWs show only the 4.7 Å signal and WT and K/R silica NWs also show signal at 10–11 Å.

**Figure 3:**
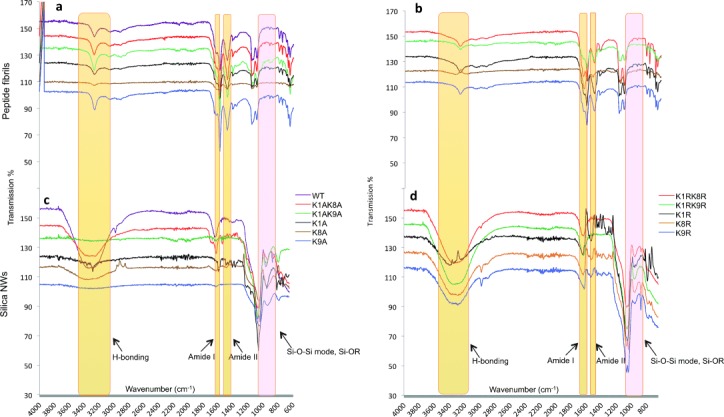
Fourier transform infrared spectroscopy reveals similarities in the spectra between the peptide fibrils and highlights the Amide I, II and hydrogen bonding bands (yellow highlights). Bands associated with silica compositions (pink highlights) are observed for the silica NWs. K1A, K9A and K1AK9A show very weak for H-bonding and Amide I and II signals. a) Shows K/A peptide fibrils and c) silica NWs. b) shows K/R peptide fibrils and d) silica NWs.

The fibrils and the silica-NWs were examined using FTIR to further explore the composition of the silica-NWs (Figure 3). Prior to silica coating, all variants showed bands consistent with hydrogen bonding between 3274–3278 cm^−1^ for K/A (Figure 3 a), and K/R (Figure 3 b). Furthermore, Amide I and II bands appeared clearly around 1624–1626 cm^−1^ and 1524–1526 cm^−1^, respectively. The position of these bands is in close agreement with C–H, O–H, Si–NH_2_, and amine N–H stretching, and NH_2_ vibration mode.[19] All Amide I bands were assigned to β-sheet structure (1637–1613 cm^−1^)[20] in agreement with X-ray FD results.

Following TEOS treatment, H-bonding bands for the K1A, K9A, and K1AK9A variants become broader and weaker. The amide bands become much weaker, and this is consistent with the observed absence of coated silica NWs observed by TEM and the absence of cross-β diffraction by X-ray FD (Figure 2). K8A and K1AK8A show broad bands for H-bonding, but rather weak signals in the amide region, whilst K1A, K9A, and K1AK9A show very weak H-bonding signals following coating (Figure 3 c). In contrast, the WT and K/R silica NWs show broad peaks in the H-bonding region. This broadening may arise from the H-bonding within the silica coating and/or to water binding.[21] All K/R silica coated fibrils also retain the strong and sharp Amide I/II peaks observed in fibrils prior to TEOS treatment. This is consistent with the peptide core being retained within the K/R silica NWs. Following TEOS treatment, all samples (K/A and K/R variants) display three characteristic silica peaks in their spectra (Figure 3 c, d); the asymmetric stretching vibrations of Si–O–Si, which can be clearly observed with strong signals at 1081–1038 cm^−1^. Bands of Si–R and –Si–OR stretching (962–945 cm^−1^ from K/A, and 970–962 cm^−1^ from K/R silica NWs) also may arise from crystalline silica structures. Finally, bands at 871–810 cm^−1^(K/A) and 893–871 cm^−1^(K/R) can be assigned to symmetric stretching vibrations of Si–O–Si (Figure 3 c, d).[22] These Si peaks were not observed in IR spectra from fibrils prior to silica coating (Figure 3 a, b).

To further explore the structure and morphology of the K/R silica NWs, the most ordered silica NW structures were embedded in resin and sectioned to enable the views of cross-sections. Figure 4 reveals that K1R and K9R show the silica NWs with electron lucent core. Cross-sections measure approximately 40–50 nm diameter.

**Figure 4:**
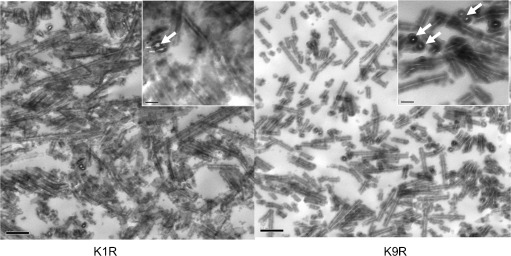
Thin sections of embedded silica NWs formed by the K1R and K9R variants. Scale bars=0.2 μm for the main images and 50 nm for the higher magnification inserts. White arrows highlight silica NW cross-sections.

All peptides explored were observed to form cross-β amyloid fibrils by TEM, and X-ray FD as previously described.[16] K/A variants were generally narrow filaments, whilst K/R variants formed laterally associated structures that appeared to be more crystalline in appearance. All the peptide fibrils were treated with TEOS, and their ability to template silica to form silica-NWs varied with the different sequences. The WT and K/R variants formed silica NWs, however, K/A formed silica NWs less readily. K1A, K1AK8A, and K9A were very inefficient in catalyzing the formation of the silica coating. It appears that the lysine at position 1 may play a particularly important role in catalysis of the templating, and K9A may also participate in this process (Figure 5 a). Both K1 and K9 lie on the same side of the β-strand, and this indicates that these residue positions lie on the outer surface of the fibrillar structures (Figure 5 b,c). Interestingly, the K1AK9A peptide formed ordered narrow fibrils and silica NWs following TEOS treatment that were observed by TEM. However, these NWs did not show β-sheet signals in FTIR or X-ray. This may suggest that K8 is able to partially participate in the silica coating, but the NWs formed are relatively unstable and do not give rise to diffraction or FTIR data similar to other NWs.

**Figure 5:**
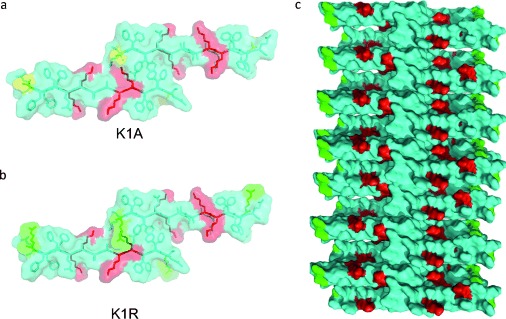
Structural models based on the original structure[15] with peptide colored cyan, lysines red, alanine yellow, and arginine green. a) and b) show the view down the fiber axis so that the interdigitation of side chains across the β-sheets can be observed whilst c) depicts the fibril axis, whereby the β-strands stack through hydrogen bonding. a) The loss of the key lysine residue in K1A leading to loss of ability to form silica NWs. b) K1R showing arginine residue in green on the edge of the peptide. c) A model of the peptide in the fibril showing the lysine and arginine residues that can template the TEOS to form silica NWs.

The role for lysine residues in catalyzing condensation of TEOS to form a silica coating has been previously described.[12, 22b] Clustering of the lysine residues has been revealed in previous work on diatoms. Silaffins contain clusters of five lysines that have been shown to promote silica templating.[23] Here, we have revealed that the sequence position of lysine is important, as well as its arrangement within the three dimensional structure.

Interestingly, in the presence of K1AK8A, silica shells or spheres were observed, so whilst coating of fibrils to form silica NWs did not occur, the peptide may still be able to catalyze formation of spherical forms of the silica. In the context of the other peptides that are able to template silica, lysine residues are found exposed and stacked up along the fibril axis, leading to the template assembly of the silica coating (Figure 5 c). Here, we show that arginine is also very capable of templating the silica assembly to form silica NWs.[24] Indeed, K9R reveals very straight silica NWs and these were narrower than those formed by K8R. This suggests that the positions of the R residues may influence the morphology of the different nanowires.

Here, we reveal how changes in amino acids sequence can influence the ability of fibrillar structures to template silica. Furthermore, we provide evidence that the peptide core of these silica nanowires remains following silica treatment. The nanowires we have created are extremely narrow and therefore very light. Previous work has revealed that the strength of silica nanowires increases with diameter.[25] The strength and potential applications will be the subject of intensive further work.

## Supporting Information

As a service to our authors and readers, this journal provides supporting information supplied by the authors. Such materials are peer reviewed and may be re-organized for online delivery, but are not copy-edited or typeset. Technical support issues arising from supporting information (other than missing files) should be addressed to the authors.

miscellaneous_information
