# Supplementary material for: Silica Nanowires Templated by Amyloid-like Fibrils
Source: Angew Chem Int Ed Engl. 2015 Oct 5;54(45):13327–31. doi: 10.1002/anie.201508415 (PMC4674975; doi:10.1002/anie.201508415)
Supplement: Supplementary file 1 — miscellaneous_information [file anie0054-13327-sd1.pdf]

Angewandte Chemie
Eine Zeitschrift der Gesellschaft Deutscher Chemiker

Supporting Information

Silica Nanowires Templated by Amyloid-like Fibrils

*Zahraa S. Al-Garawi, Julian R. Thorpe, and Louise C. Serpell**

anie_201508415_sm_miscellaneous_information.pdf

SUPPORTING INFORMATION

Experimental Section

*Peptide preparation***.**

NH_2-KFFEAAAKKFFE-COOH variant peptides were synthesised using FMOC chemistry at 95% purity and were a gift from Dr. Helen Walden (Cancer research UK), and are shown in Table 1. Stock solutions (10 mg/ml) of peptides were prepared by solubilising the lyophilised peptide in filtered, milli-Q water, or phosphate buffer saline (PBS), pH 7.4. WT peptide and all K/A variants were dissolved in water except for K1A, which was dissolved in PBS, and all K/R variants in PBS. After dissolving, the peptides were vigorously vortexed for 1 minute, spun at 13000 rpm for 5 min. and any undissolved particles were discarded. Peptides were incubated for seven days at room temperature (RT) to allow self-assembly and biophysical characterizations were conducted immediately (zero time), and after seven days [16].

Supplementary Table 1. Peptide sequences.

K/A variant	Sequence	K/R variant	Sequence
WT	KFFEAAAKKFFE		
K1A	**A**FFEAAAKKFFE	K1R	**R**FFEAAAKKFFE
K1AK8A	**A**FFEAAA**A**KFFE	K1RK8R	**R**FFEAAA**R**KFFE
K1AK9A	**A**FFEAAAK**A**FFE	K1RK9R	**K**FFEAAAK**R**FFE
K8A	KFFEAAA**A**KFFE	K8R	KFFEAAA**R**KFFE
K9A	KFFEAAAK**A**FFE	K9R	KFFEAAAK**R**FFE

Preparation of Silica-NWs: Peptide variants were incubated in water or phosphate buffered saline (PBS) for over seven days to ensure formation of fibrils and then a cold solution (2ml) of TEOS was added to 500 µl of the peptide solution (5mg/ml in 50 mM tris-HCl buffer, pH 6.8) to a final concentration of TEOS of 2 mM, mixed well and agitated vigorously by vortex for one minute. The resulting two-layer solution was then allowed to settle without stirring. After 36 h, a white precipitate appeared in the aqueous phase, which was collected by centrifugation (14000 rpm, 3 min.), washed with annular ethanol 99% three times [26], dissolved in 125 µl filtered milli-Q water, and then used for biophysical and imaging analysis. To further wash the NWs, they were treated with 1M NaOH after washing with ethanol, and then dissolved in water prior to Xray FD.

Negative stain transmission electron microscopy (TEM): The 10 mg/ml stock was diluted to 5 mg/ml and 4 µl of fibril solution was placed onto grids (400 mesh copper grid with Carbon/Formvar film from Agar Scientific) and incubated for two minutes, followed by a two minute wash with filtered milli–Q water (4 µl), and then negatively stained using 2% w/v uranyl acetate (4 µl) for two minutes. After each incubation, excess liquid was blotted using filter paper. Grids were examined using a Hitachi 7100 TEM operated at 100 kV accelerating voltage, and digital images were acquired with a Gatan Ultrascan 1000 (2K X 2K pixel) CCD camera (Gatan, Inc., Pleasanton, USA).

Thin sectioning and electron microscopy of Silica-NWs:

Dry samples of selected Silica-NWs K/R were incubated in TAAB low viscosity resin (TAAB Laboratories Equipment Ltd, Aldermaston, UK). They were left for 24h at room temperature to infiltrate with the resin before polymerising overnight at 60°C. Thin (c. 90 nm) sections were cut on a Leica Ultracut ultramicrotome (Leica Microsystems [UK] Ltd., Milton Keynes, UK), collected upon TEM support grids and stained with 0.5% (w/v) uranyl acetate for 1h. Thin sections were examined in a Hitachi 7100 TEM operated at 100 kV accelerating voltage, and digital images were acquired with a Gatan Ultrascan 1000 (2K X 2K pixel) CCD camera (Gatan, Inc., Pleasanton, USA).

X-ray Fibre Diffraction (FD) :

Ten µl of each peptide stock solution (10 mg/ml) before and after siliconisation was placed between two wax-tipped glass capillaries and allowed to align at room temperature. Aligned specimens were placed onto a goniometer head and data were collected using a Rigaku rotating anode source (CuK□) and Saturn CCD detector with specimen to detector distances of 50 or 100 mm. The diffraction patterns were examined using Mosflm [27] and CLEARER [28]. To confirm fibre alignment between capillaries, each sample was prepared twice to insure alignment.

Fourier Transform Infrared spectroscopy (FT-IR):

Powder samples of fibrillar peptides were produced by washing the white precipitate with ethanol and then drying under $N_{2\,(g)}$. Powder samples of variants before and after silica coating were then placed into the sample holder of FTIR-ATR instrument

(Perkin Elmer Spectrum one), which is inserted into the photoacoustic cell which is then sealed for FTIR measurement. Air was used as a control.

Results

Supplementary Table 2. Approximate widths of fibrils and silica NW.

Widths (nm)	WT	K1A	K1AK8A	K1AK9A	K8A	K9A	K1R	K1RK8R	K1RK9R	K8R	K9R
**Before silica**	11.7	16.2	20.6	10.7	8.8	9.8	18.6	26.6	21.1	17.5	22.8
**Silica NW Bundles**	78.3	**-**	**-**	65.5	65.7	**-**	48.5<br>776	9.6<br>558	69.7	90<br>238	62.2

Supplementary Table 3. Comparison between K/A and K/R silica NWs.

K/A variant	Description	K/R variant	Description
WT	Few, straight wires		
K1A	Very few	K1R	Many, variable straight bundles some amorphous.
K1AK8A	Amorphous, no NTs	K1RK8R	
K1AK9A	Many, straight, short and ordered individuals and bundles	K1RK9R	Wide, long, single<br>Few, bulky wires
-K8A	Many lateral bundles, straight and ordered.	K8R	Many, very wide, straight, lateral bundles.
K9A	Very few, wide and flexible	K9R	Many very straight ordered NTs
